# *Escherichia coli* STb Enterotoxin Dislodges Claudin-1 from Epithelial Tight Junctions

**DOI:** 10.1371/journal.pone.0113273

**Published:** 2014-11-19

**Authors:** Hassan Nassour, J. Daniel Dubreuil

**Affiliations:** GREMIP, Faculty of Veterinary Medicine, Université de Montréal, Montreal, Quebec, Canada; Emory University School of Medicine, United States of America

## Abstract

Enterotoxigenic *Escherichia coli* produce various heat-labile and heat-stable enterotoxins. STb is a low molecular weight heat-resistant toxin responsible for diarrhea in farm animals, mainly young pigs. A previous study demonstrated that cells having internalized STb toxin induce epithelial barrier dysfunction through changes in tight junction (TJ) proteins. These modifications contribute probably to the diarrhea observed. To gain insight into the mechanism of increased intestinal permeability following STb exposure we treated human colon cells (T84) with purified STb toxin after which cells were harvested and proteins extracted. Using a 1% Nonidet P-40-containing solution we investigated the distribution of claudin-1, a major structural and functional TJ protein responsible for the epithelium impermeability, between membrane (NP40-insoluble) and the cytoplasmic (NP-40 soluble) location. Using immunoblot and confocal microscopy, we observed that treatment of T84 cell monolayers with STb induced redistribution of claudin-1. After 24 h, cells grown in Ca^++^-free medium treated with STb showed about 40% more claudin-1 in the cytoplasm compare to the control. Switching from Ca^++^-free to Ca^++^-enriched medium (1.8 mM) increased the dislodgement rate of claudin-1 as comparable quantitative delocalization was observed after only 6 h. Medium supplemented with the same concentration of Mg^++^ or Zn^++^ did not affect the dislodgement rate compared to the Ca^++^-free medium. Using anti-phosphoserine and anti-phosphothreonine antibodies, we observed that the loss of membrane claudin-1 was accompanied by dephosphorylation of this TJ protein. Overall, our findings showed an important redistribution of claudin-1 in cells treated with STb toxin. The loss of phosphorylated TJ membrane claudin-1 is likely to be involved in the increased permeability observed. The mechanisms by which these changes are brought about remain to be elucidated.

## Introduction

Enterotoxigenic *Escherichia coli* (ETEC) represent an important cause of severe diarrhea in newborn animals [1] and diarrhea in humans following the ingestion of contaminated food and water [2]. Expression of both colonization factors and toxins are required for disruption of intestinal fluid homeostasis, leading to diarrhea [3]. ETEC strains are known to produce several types of enterotoxins, including heat-labile enterotoxin (LT), heat-stable enterotoxin a (STa) and heat-stable enterotoxin b (STb) [4]. Enteroaggregative heat-stable toxin 1 (EAST1) was also shown to be produced by ETEC [5], [6].

STb, a 48-amino-acid peptide of 5.2 kDa, secreted by ETEC strains is mainly associated with post-weaning diarrhea in piglets [7], [8]. *In vivo*, STb binds to its receptor, sulfatide, an acidic glycosphingolipid localized at the surface of intestinal epithelial cells [9]. Then, STb is internalized and stimulates a pertussis toxin-sensitive G protein (Gαi3) [10]. This causes an influx of extracellular calcium ions through a ligand-gated calcium ion channel. The increased intracellular Ca^++^ stimulates protein kinase C (PKC) that phosphorylates and activates the cystic fibrosis transmembrane regulator (CFTR), leading to Cl^-^ secretion. The calcium increase also activates phospholipases A_2_ responsible for the release of arachidonic acid from membrane phospholipids leading to production of prostaglandin E_2_ (PGE_2_) and 5-hydroxytryptamine (5-HT) [11], [12]. These molecules mediate transport of water and HCO_3_^-^ from enterocytes into the intestinal lumen and prevent Na^+^ absorption resulting in watery diarrhea [7].

Using immunoblot or enzyme-linked assays, Berberov et al. (2004) demonstrated that EAST-1, LT and STb could be concurrently expressed by porcine ETEC strains [13]. Also, Zhang et al. (2006) observed that only LT-and STb-positive strains caused appreciable diarrhea in 5-days-old pigs [14]. Futher, Erume et al. (2013) results indicated that STb is a more significant contributor to diarrhea for weaned pigs [15]. In the same way, recent data by Loos et al. (2012) suggested a dominant role for STb in small intestinal secretion early after post-weaning infection, as well as in the induced innate immune response through differential regulation of immune mediators like interleukin-1 and interleukin-17 [16].

The intestinal lumen is covered by a uniform single layer of epithelial cells. This epithelial layer serves as an environmental barrier [17]. This barrier comprises a number of transmembrane proteins including, but not limited to, tight junction (TJ) proteins like occludins, claudins, and junctional adhesion molecules (JAMs). TJ-associated proteins includes cytoplasmic peripheral membrane proteins such as ZO-1 and ZO-2 known to be associated with transmembrane proteins that includes occludin and claudin families with the apical perijunction of F-actin ring [18]. The F-actin network forms the cell cytoskeleton [19]. TJs act as a barrier controlling penetration of ions, solutes, and water, through intercellular spaces and act as a fence dividing apical and basolateral domains to compartmentalize the plasma membrane. These characteristics of TJs provide also a barrier to prevent the entry of pathogens and foreign substances from invading and facilitate directional exchanges of material [20]. Besides the cell maintenance TJs are essential to both cellular development and normal barrier function [21].

Although human epithelial cells have incorporated barriers to block microorganisms to gain access to deeper cell layers within tissues, certain pathogens have evolved to exploit and thus control TJs to alter this barrier. These pathogens use an array of tactics to hijack junctional structures to their advantage. Some pathogens use TJ proteins as receptors for attachment and subsequent internalization. Others destroy the TJs thereby providing a gateway to the underlying tissue. For example, pathogens such as enteropathogenic *E. coli*, serotype O127:H6 [22], enterohemorrhagic *E. coli* producing a shiga toxin-independent non-bloody diarrhea [23], serotype O157:H7 [24], bacterial toxins such as *Clostridium difficile* toxins A and B [25], *Vibrio cholerae* Zonula occludens toxin [26], and *E. coli* secreted autotransporter toxin [27] disrupt TJs [28].

Many studies on ETEC enterotoxins used T84 human colon cells, a cell line commonly used to study bacterial enterotoxin secretory processes [40]. Kreisberg et al. (2011) observed that LT-producing strains could affect cellular permeability independently of STa production [29]. However, in a study of Nakashima et al. (2013), STa elicited a reduction in TER and causes not only induction of water secretion but also intestinal barrier dysfunction but did not increase the paracellular permeability to FITC-labelled dextran [30]. For STb toxin, a reduction in TER associated with an increased in paracellular permeability was associated with a marked alteration of F-actin stress fibers [31]. F-actin filament dissolution and condensation were accompanied by redistribution and/or fragmentation of ZO-1, claudin-1, and occludin. Therefore, reduction in TER resistance and paracellular permeability to FITC-labeled dextran is recognized as indices of the decreased integrity of epithelial cells intoxicated with these toxins.

In a recent study, STb toxin generated an increase in cytoplasmically located TJ proteins including claudin-1 [31]. Less phosphorylated claudin-1 is found in the cytoplasm and highly phosphorylated claudin-1 is selectively concentrated at TJs monitored as NP-40-insoluble material [28], [32], [33]. Detergent insolubility of proteins is considered to indicate their integration into macromolecular phosphorylated complexes such as intercellular junctions [32], [34]. Membrane-associated claudin-l is known to be important structural and functional components in maintaining TJ integrity [35].

PKC, a family of serine-threonine kinases, are known to regulate epithelial barrier function. PKC are epithelial calcium-dependent enzymes and appears to regulate both subcellular localization and phosphorylation states of several TJ-associated proteins including claudin-1 [36]. The aim of the present study was to examine the effects of STb on location and phosphorylation state of claudin-1 in T84 intestinal epithelial cells.

## Materials and Methods

### Culture media, antibodies, and reagents

Dulbecco’s modified Eagle medium (DMEM), Ham’s F-12 nutrient mixture (F-12), phosphate-buffered saline (PBS; pH 7.4, free of calcium chloride and magnesium chloride), 5% fetal bovine serum (FBS), rabbit polyclonal anti-claudin-1, goat anti-rabbit Alexa 488 antibodies, bovine serum albumin (BSA), and DAPI (4′,6-diamidino-2 phenylindole dihydrochloride) were purchased from Invitrogen. FITC-phalloidin and Isopropyl-ß-D-thiogalactopyranoside (IPTG) was purchased from Sigma. Factor Xa was from Roche and phenymethanesulfonyl fluoride (PMSF) was from Gibco BRL. Anti-phosphoserine and anti-phosphothreonine antibodies were purchased from Abcam.

### Production and STb purification

Recombinant STb toxin was produced as described previously, using a HB101 strain harboring the plasmid pMAL-STb, which codes for the fusion protein MBP-STb [37]. Ampicillin, at a final concentration of 50 mg/ml, was used as the selection agent for bacteria carrying the plasmid pMAL-STb. Bacteria were grown in Rich medium (10 g tryptone, 5 g yeast extract, 5 g NaCl, 2 g dextrose per liter) for 18 h at 37°C in an orbital shaker set at 180 rpm. A volume of 5 ml of an overnight bacterial culture was transferred to 500 ml of fresh Rich medium and returned to the orbital shaker until the absorbance at 600 nm reached 0.5. Then, 0.3 mM IPTG was added to induce the synthesis of the fusion protein. The induction was allowed to proceed for 3 h in the orbital shaker. Cells were harvested by centrifugation at 4,000×g for 15 min at 4°C. The pellet was gently washed in a volume of 250 ml of 30 mM Tris-HCl (pH 8.0) containing 20% sucrose and 1 mM EDTA. After centrifugation at 8,000×g for 20 min at 4°C, an osmotic shock of bacteria was induced using a solution of 5 mM MgSO_4_ containing 0.4 mM PMSF and then centrifuged at 7,000 g for 20 min at 4°C. The supernatant containing the fusion protein (MBP-STb) was filter-sterilized using a 0.22-µm-pore-size tangential flow filter (VacuCap; Pall Life Sciences). The fusion protein was affinity-purified at 4°C on a 30 ml amylose column (New England BioLabs) at a flow rate of 0.2 ml/min using a column buffer solution of (10 mM Tris-HCl (pH 7,5), 200 mM NaCl, 1 mM (pH 8.0)). Then, 10 mM of maltose was added to this buffer in order to elute maltose-binding proteins. The eluted proteins were dialyzed against MilliQ water using a 12,000 to 14,000 Da membrane (Spectrum). Dialyzed material was then concentrated using a speed-vac and cleaved using factor Xa enzyme (Roche) in a cleavage buffer consisting of 50 mM Tris-HCl, 100 mM NaCl, and 1 mM CaCl_2_ (pH 8.0). Using an AKTA-10 purifier system (GE Healthcare), the cleaved material was loaded onto a C_8_ reverse-phase column (PerkinElmer) and eluted with a linear gradient of acetonitrile in water solution containing 0.1% trifluoroacetic acid. Using a Nanodrop ND-1000 spectrophotometer (Thermo Scientific), a standard curve of various concentrations of aprotinin (molecular weight of ∼6,500) with absorbance at 214 nm was prepared. STb preparations were quantified using the generated standard curve and kept at −20°C until use [38].

### Intestinal cell culture and treatments

T84 human colon intestinal epithelial cells, used as a model to study the effects of enterotoxins on TJ proteins [29], [30], [39]–[42] were obtained from the American Type Culture Collection. Cells (passage 5 to 18) were maintained in equal volumes of Dulbecco’s Modified Eagle Medium (DMEM) and F-12 supplemented with 5% (vol/vol) fetal bovine serum (FBS) (Invitrogen). Cells were grown in T-75 culture flasks (Sarstedt) at 37°C, 5% CO_2_ in a humidified incubator. Cell viability of T84 cell line was measured using Trypan blue [43]. For maintenance purposes, confluent T84 monolayers were passaged weekly using trypsin-EDTA treatment in phosphate buffered saline (PBS: Invitrogen) free of calcium chloride and magnesium chloride.

For immunofluorescence experiments, T84 cells were seeded, on LabTek 8-well chamber slides (Fisher Scientific), at a density of 150,000 cells/ml and used after 2 days of growth. One hour before treatment with purified STb cell monolayers was washed using PBS and the medium was changed to medium without FBS.

### Detergent extraction of cell monolayers

Cell monolayers were grown on tissue culture plates and treated with purified STb (4 nmoles added to 9 ml of cell culture medium) for 0, 6, 12 and 24 h and then subjected to detergent extraction with non-ionic Nonidet P-40 (a non-ionic, non-denaturing detergent) (NP40) according to the method of Sakakibara et al. (1997) [32]. After STb treatment, cells were washed three times with cold PBS, and centrifuged for 10 minutes at 16 000×g. After centrifugation, the cell pellet was lyzed with ice-cold NP-40 buffer (25 mM HEPES/NaOH (pH 7,4), 150 mM NaCl, 4 mM EDTA, 25 mM NaF, 1% NP-40, 1 mM Na_3_VO_4,_ 1 mM PMSF, 10 µg/ml leupeptin, 10 µg/ml aprotinin). After centrifugation at 16 000×g, the supertant was collected as the NP40-soluble fraction. The pellet was resuspended in sodium dodecyl sulfate (SDS) lysis buffer (1% SDS, 25 mM HEPES (pH 7.4), 4 mM EDTA, 25 mM NaF) and homogenized. The homogenate was diluted with an appropriate volume of NP40 buffer and the lysate was left for 10 minutes and recentrifuged. The supernatant was collected and used as the NP-40-insoluble fraction.

### Gel electrophoresis and immunoblotting

For electrophoresis, equal amounts of total protein within each fraction were loaded onto a polyacrylamide gel. Proteins were dissolved in sample buffer (10% glycerol, 5% β-mercaptoethanol, 3% SDS, 0.0625 M Tris-HCl (pH 6.8), 0.01% bromophenol blue) and heated for 10 minutes at 100°C. Samples were resolved by one-dimensional SDS-PAGE as described by Laemmli (1970) using a 12% gel and the fractioned proteins were electroblotted onto a PVDF membranes (Millipore, 0,45 µm pore size) [44]. After blocking the membrane with 3% milk in PBS containing 0.1% Tween 20 (PBS-T), membranes were incubated with polyclonal antibodies against claudin-1 (1∶2000) (Invitrogen), phosphothreonine and phosphoserine (1∶2000) (Abcam), washed in PBS-T and further incubated with HRP-conjugated secondary antibodies (1∶4000) (Biorad). A liquid substrate system for membranes TM-blue (Sigma) was used to detect the enzymatic activity of the secondary antibody. The density ratio of the specific bands was quantified using ImageJ (National Institutes of Health, Bethesda, MD). Representative blots from multiple experiments (minimum of three) are shown in the figures. Blots were digitally contrasted to preserved relative intensity of specific claudin-1 bands.

### Cations enrichment studies

T84 cells were grown in calcium-free medium (Dulbecco’s Modified Eagle Medium (DMEM), Ham’s F-12 nutrient mixture (F-12) with 5% FBS) and then transferred to the calcium-enriched medium (1.8 mM CaCl_2_) for 24 h and then treated with 4 nmoles of purified STb for 0, 6, 12 and 24 h. Cells were then subjected to detergent extraction, resolved by one-dimensional SDS-PAGE and electrophoretically transferred as described above. The same experiments were done using two others divalent ions, Mg^++^ and Zn^++^, to assess the specificity of Ca^++^ in the increased rate of claudin-1 dislodgement and actin disorganization.

### Immunofluorescence microscopy of actin cytoskeleton and tight junction proteins

For claudin-1 staining, T84 cells were seeded on cover slips at 1×10^5^ cells/ml and fixed with 100% ethanol for 20 minutes. Cells were then permeabilized with 1% Triton X-100 in PBS for 10 minutes, soaked in blocking solution (PBS containing 1% BSA) for 1 h, and then incubated with the anti-claudin-1 antibody (1∶100) for 1 h in a moist chamber. The samples were washed three times with PBS and then incubated for 1 h with FITC-conjugated goat anti-rabbit IgG (Biorad). Samples were then washed with PBS three times, mounted in PBS containing 1% p*-*phenylenediamine and 90% glycerol.

For staining of F-actin filaments, cells were washed three times with PBS, permeabilized with 0.1% Triton X-100 for 10 min at room temperature, and blocked with 2% BSA–PBS for 45 min before incubation with fluorescein-phalloidin for 45 min at room temperature [31]. The cell monolayers were washed again three times with PBS. The slides were observed with a confocal microscope (Olympus FV1000IX81) at x40 magnification for claudin-1 and F-actin.

### Statistical analyses

For claudin-1 dislodgment study in Ca^++^-free medium, a linear mixed model was used with treatment as a fixed factor and id (replicated unit) as a random factor to account for the lack of independence among the three samples for each subject. Tukey’s post-hoc tests were used to examine the differences between pairs of treatment means.

For divalent-enriched cations experiments, data were transformed using the logarithm base 10 to normalize distributions. A linear mixed model was used with treatment and time as fixed factors and sample id as a random factor. A priori contrasts were performed to compare pairs of means adjusting the alpha level for each comparison using the Bonferroni sequential procedure to maintain the experiment error rate at the nominal value. Statistical analyses were carried out with SAS v.9.3 (Cary, N.C.) and the level of statistical significance was set at 5% throughout.

## Results

### STb affects the distribution of claudin-1

**Figure 1:**
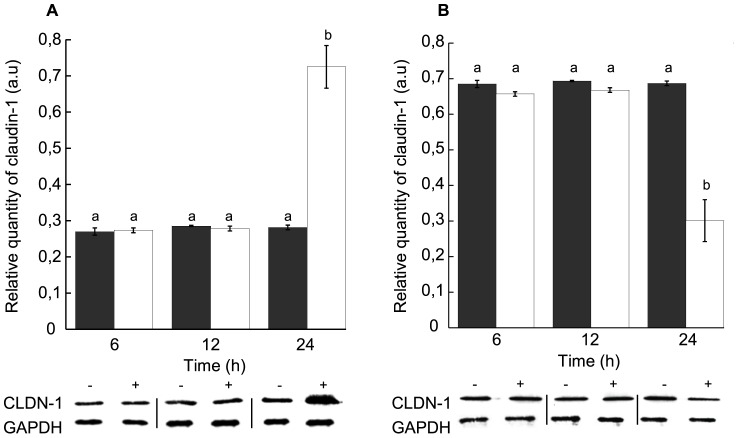
Distribution of claudin-1 in Nonidet P-40-soluble and -insoluble fractions in T84 cell monolayers in response to STb toxin. (A) NP-40-soluble and (B) NP-40-insoluble fractions. Black: untreated cells, white: cells treated with STb for various times. Lower panel: Immunoblot showing claudin-1 and GAPDH used to evaluate their relative amounts. Proteins were separated on a 12% acrylamide SDS-PAGE and immunoblotted with appropriate antibodies. After 24 h, STb treatment induced a loss of claudin-1 in the NP-40-insoluble fraction (membrane location). At the same time, a comparable increase of claudin-1 was observed in the NP-40-soluble fraction (cytoplasm location) (n = 3) (p<0.001). However, no differences were observed between STb-treated and -untreated cell monolayers after 6 or 12 h treatment. CLDN-1: claudin-1; GAPDH: Glyceraldehyde 3-phosphate dehydrogenase. Letters on top of the bars when different indicates a statistical difference between the treatments. The minus and plus signs over the immunoblots indicate respectively that cells were not treated or treated with STb toxin.

Since STb toxin was previously shown to increase paracellular permeability [31], we wanted to address whether translocation of TJ proteins was involved in this process. We thus analyzed the time-dependent distribution of claudin-1, a protein with a major role in sealing TJs, between Nonidet P-40-soluble and -insoluble fractions (Fig. 1).

Purified STb toxin (4 nmoles) was added to the apical side of T84 cell monolayers, and incubated for 6, 12 or 24 h. Cell proteins were then extracted with a buffer containing Nonidet P-40 to determine the amount of membrane-associated claudin-1 (NP-40-insoluble) and cytoplasmically located (NP-40-soluble). Extracted proteins were separated on a 12% acrylamide SDS-PAGE and western blotted using an anti-claudin-1 antibody. The relative intensity of claudin-1 protein bands was measured and compared to the intensity of GAPDH, our internal control. The negative control consisted of untreated cell monolayer.

In STb treated cells, we observed, after 24 h, a marked increase in the level of NP-40-soluble claudin-1 and a decrease in NP-40-insoluble claudin-1 (Fig. 1). Densitometric analyses revealed a 40% increase in NP-40-soluble claudin-1 level and a comparable decrease in the NP-40- insoluble level. There were no significant differences in claudin-1 amount between untreated cell monolayers and cell monolayers treated with STb for 6 h or 12 h. After 24 h, the difference in the distribution of claudin-1 was statistically significant compared to the control.

**Figure 2:**
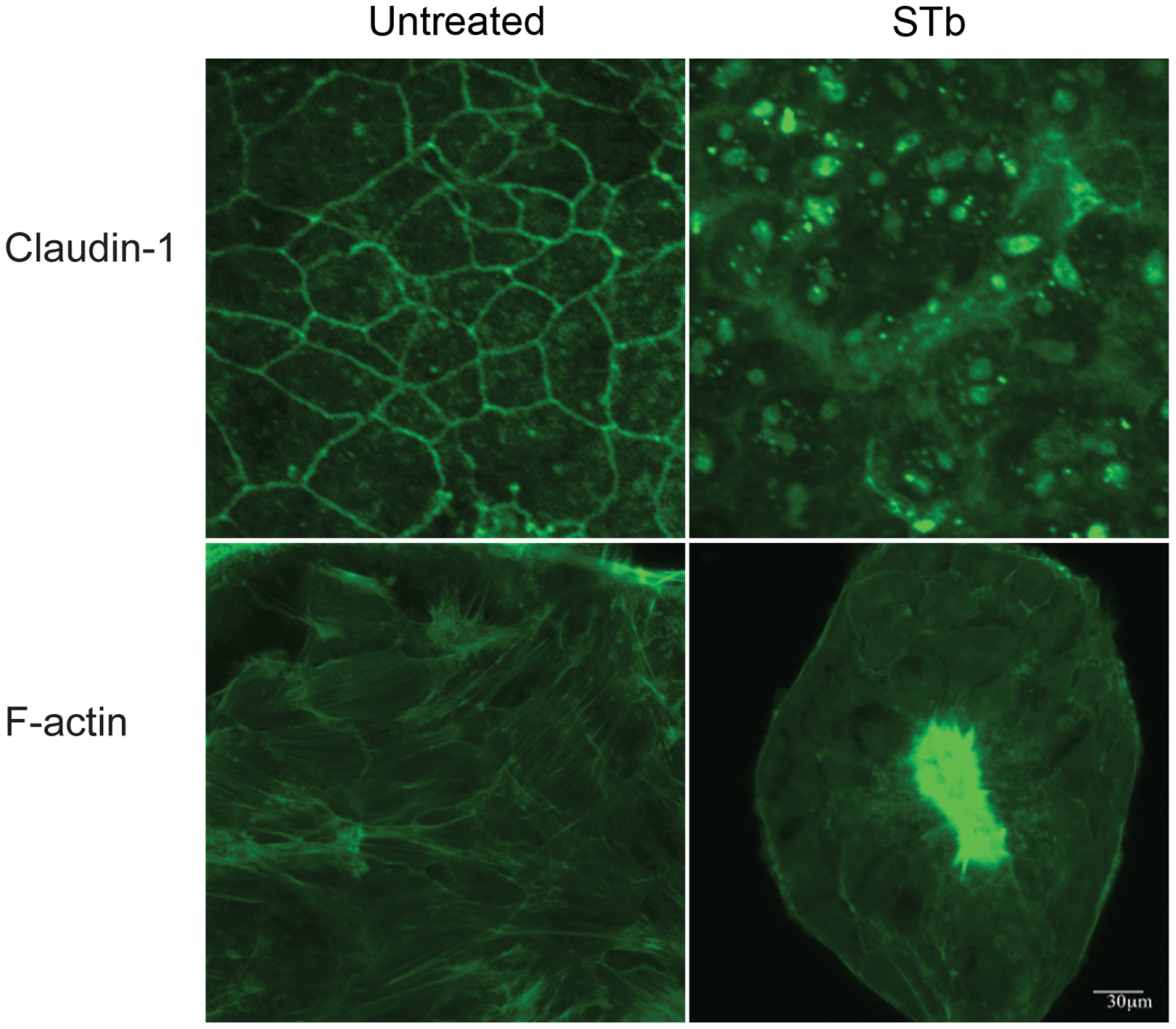
Effect of STb on claudin-1 and F-actin arrangement. After 24 h treatment with STb, T84 cells were fixed, permeabilized, and incubated with anti-claudin-1 antibody. Secondary antibody coupled to Alexa 488 was then added. F-actin was stained using FITC-phalloidin. Untreated cell monolayers stained for claudin-1 showed continuous bands around each cell whereas STb toxin provoked a loss of the “chicken wire” pattern with focal grouping. In untreated cell monolayers, regularly arranged actin filaments around cell contour and as stress fibers were observed. In STb-treated cells, condensation of the F-actin was observed. Three independent experiments were conducted, and representative images are shown (p<0.001). Bar, 30 µm.

Also, following a 24 h treatment with STb, T84 cell monolayers were fixed, permeabilized, and stained to highlight the F-actin and claudin-1 organization. Using confocal microscopy, untreated T84 cell monolayers showed well-organized F-actin filaments surrounding the cells and as stress fibers and claudin-1 proteins surrounding cell boundaries while STb-treated cell monolayers exhibited a loss of organization and focal grouping of claudin-1 with condensation of F-actin filaments (Fig. 2).

### Effect of Ca^++^ on the rate of claudin-1 redistribution

Calcium plays a critical role in the regulation of several cellular processes. As Ca^++^ was previously related to STb toxicity [10] and a calcium enrichment experiment conducted by Sakakibara et al. (1997) revealed that TJ formation was accompanied by an increase in NP-40-insoluble TJ proteins levels including claudin-1 [32], this divalent metal ion was investigated in relation to its possible involvement in claudin-1 distribution.

**Figure 3:**
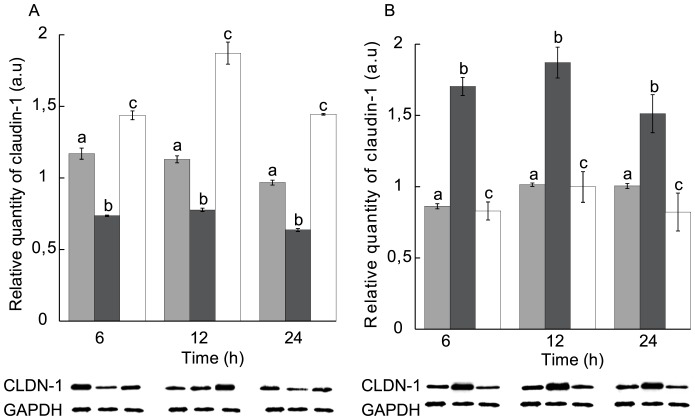
Distribution of claudin-1 in NP-40-soluble and -insoluble fractions in T84 cell monolayers in response to STb toxin after Ca^++^ enrichment. (A) NP-40-soluble and (B) NP-40-insoluble fractions. Gray: calcium-free medium, black: calcium-enriched medium, white: calcium-enriched medium treated with STb. Lower panel: Immunoblot showing claudin-1 and GAPDH used to evaluate their relative amounts. T84 cells were grown in calcium-enriched medium (1.8 mM) for 24 h and treated with STb toxin for 6, 12 or 24 h. Cells were extracted with Nonidet P-40 buffer. In Ca^++^-enriched medium, the amount of NP-40-insoluble claudin-1 was increased compared to calcium-free condition. Addition of STb toxin provokes dislodgment of NP-40-insoluble claudin-1 to the soluble fraction (n = 3) (p<0.001). CLDN-1: claudin-1; GAPDH: Glyceraldehyde 3-phosphate dehydrogenase. Letters on top of the bars when different indicates a statistical difference between the treatments.

We thus examined the relation between calcium, STb toxin and NP-40-insoluble claudin-1 level. First, in calcium-free medium in absence of STb, claudin-1 was approximately equally distributed between the NP-40-soluble and -insoluble fractions (Fig. 3A and B; gray bar). However, after calcium enrichment (1.8 mM CaCl_2_), the bulk of claudin-1 was found in the NP-40-insoluble fraction (Fig. 3B; black bar). Incubation of T84 cells in calcium-enriched medium in presence of STb resulted in a significant dislodgement of claudin-1 observed as an increase in NP-40-soluble claudin-1 (Fig. 3A and B; white bar). For STb-treated cells, in Ca^++^-enriched medium, changes in the distribution of claudin-1 were apparent after 6 h and were similar after 12 and 24 h (Fig. 3; white bar). Overall, compared to GAPDH, the amount of claudin-1 was similar under the various conditions tested. Only the distribution between membrane and cytoplasmic locations was affected.

**Figure 4:**
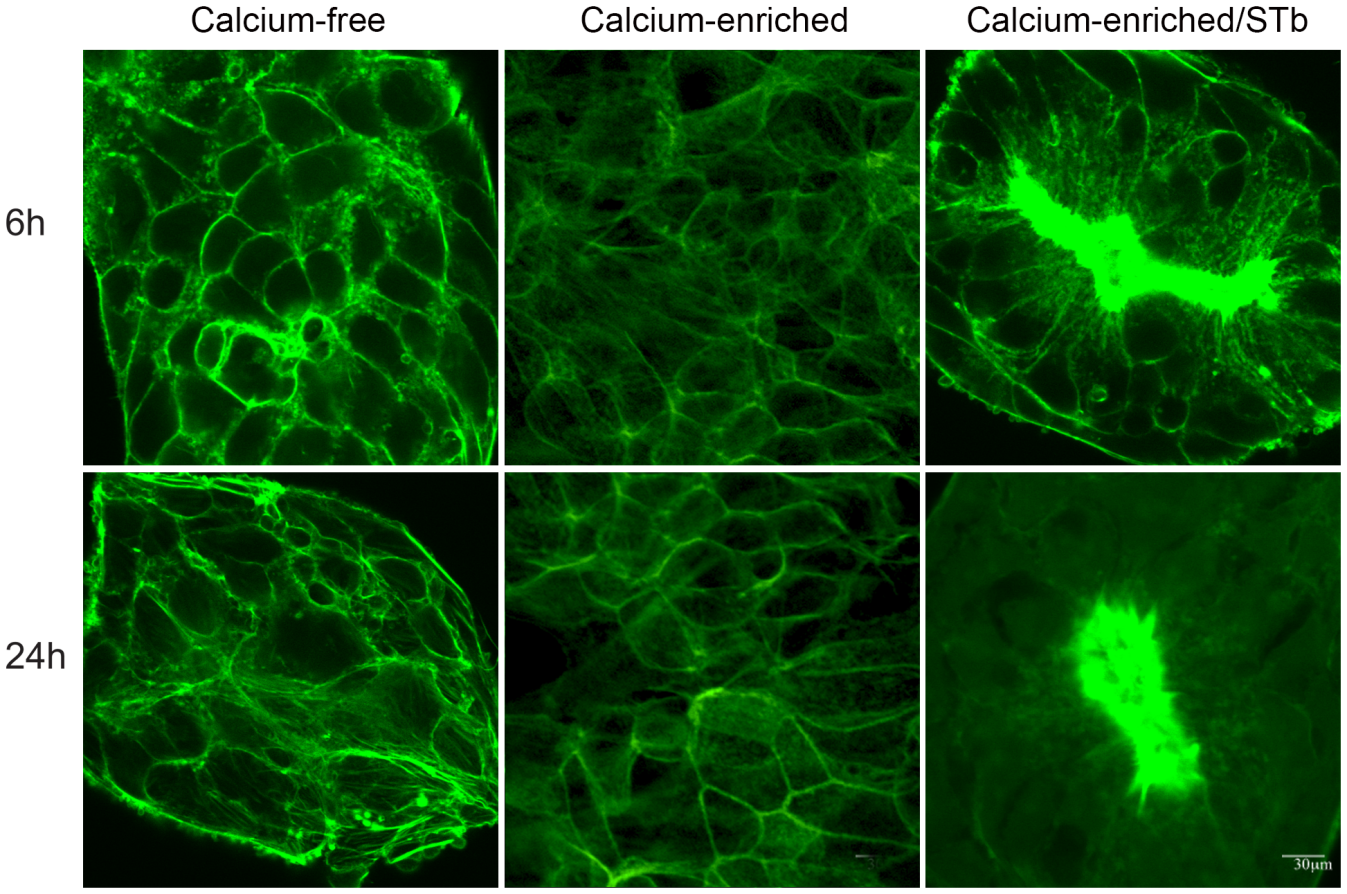
Effect of Ca^++^-enrichment on the rate of STb toxin activity. Cells grown in calcium-free and calcium-enriched (1.8 mM) media were compared after 6 and 24 h. Confocal microscopy was used to analyze the distribution of actin filaments stained with FITC-phalloidin. Calcium-enriched medium had no visible effect on the actin organization whereas in calcium-enriched medium STb provoked actin condensation after 6 h. In calcium-free medium, actin condensation was observed only after 24 h (Data not shown) Bar, 30 µm.

To examine how STb in presence of calcium influenced cell morphology, confluent T84 cell monolayers were incubated for 6 h and 24 h. Confocal microscopy analyses indicated that after 6 and 24 h, calcium-enrichment alone had little effect on actin filament organization. In fact, the amount of F-actin detectable at the edges of the cells was lowered. This can be related to the actin rearrangement that is expected when shifting from calcium-free to calcium-enriched medium. Cells grown in calcium-free and calcium-enriched media still showed well-organized F-actin filaments circling each cell as well as stress fibers (Fig. 4; calcium-free and calcium-enriched). However, after 6 h in calcium-enriched medium, STb-treated cell monolayers exhibited disruption of actin filaments surrounding the cells and stress fibers with condensation of F-actin filaments (Fig. 4; calcium-enriched/STb). In calcium-free medium, actin condensation was not observed before 24 h in presence of STb toxin (Data not shown).

### Effect of Mg^++^ and Zn^++^ enrichment

**Figure 5:**
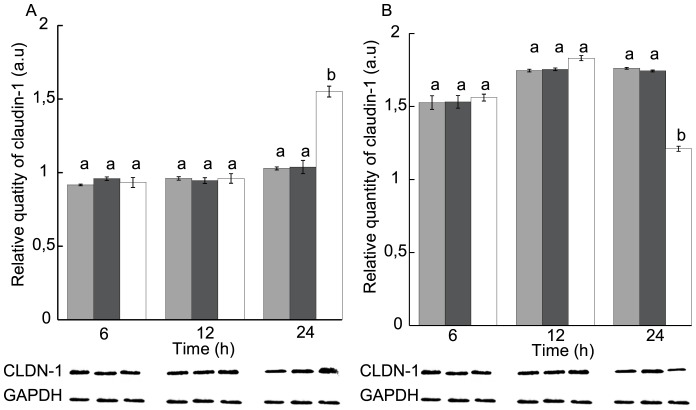
Effect of Mg^++^-enrichment on claudin-1 displacement rate. (A) NP-40-soluble and (B) NP-40-insoluble fractions. Gray: calcium-free medium, black: Mg^++^-enriched medium, white: Mg^++^-enriched medium treated with STb for 6, 12 and 24 h. Lower panel: Immunoblot showing claudin-1 and GAPDH used to evaluate their relative amounts. NP-40 cell extracted proteins were separated on a 12% acrylamide SDS-PAGE and immunoblotted with anti-claudin-1 and anti-GAPDH antibodies. The calcium-free medium was Mg^++^ -enriched (1.8 mM). There was no significant difference in claudin-1 dislogment rate under Mg^++^-enriched condition compared to calcium-free medium. After 24 h, claudin-1 dislodgement was observed as seen before in calcium-free medium (n = 3) (p<0.001). CLDN-1: claudin-1, GAPDH: Glyceraldehyde 3-phosphate dehydrogenase. Letters on top of the bars when different indicates a statistical difference between the treatments.

**Figure 6:**
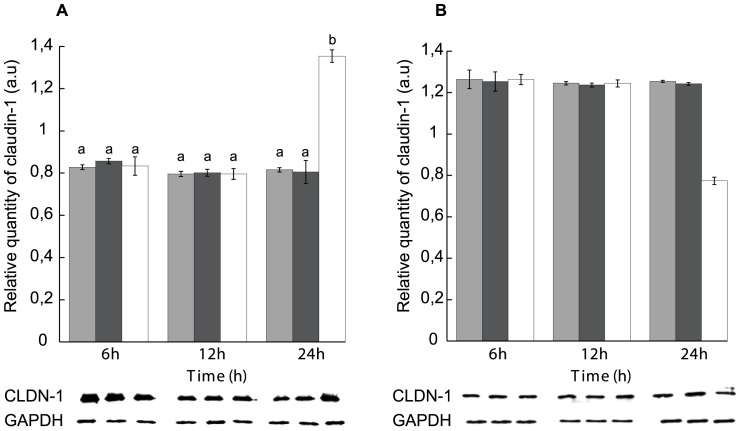
Effect of Zn^++^ -enrichment on claudin-1 displacement rate. (A) NP-40-soluble and (B) NP-40-insoluble fractions. Gray: calcium-free medium, black: Zn^++^-enriched medium, white: Zn^++^-enriched medium treated with STb for 6, 12 and 24 h. Lower panel: Immunoblot showing claudin-1 and GAPDH used to evaluate their relative amounts. NP-40 cell extracted proteins were separated on a 12% acrylamide SDS-PAGE and immunoblotted with anti-claudin-1 and anti-GAPDH antibodies. The calcium-free medium was Zn^++^ -enriched (1.8 mM). There was no significant difference in claudin-1 dislogment rate under Zn^++^-enriched condition compared to calcium-free medium. After 24 h, claudin-1 dislogment was observed as seen before in calcium-free medium (n = 3) (p<0.001). CLDN-1: claudin-1, GAPDH: Glyceraldehyde 3-phosphate dehydrogenase. Letters on top of the bars when different indicates a statistical difference between the treatments.

**Figure 7:**
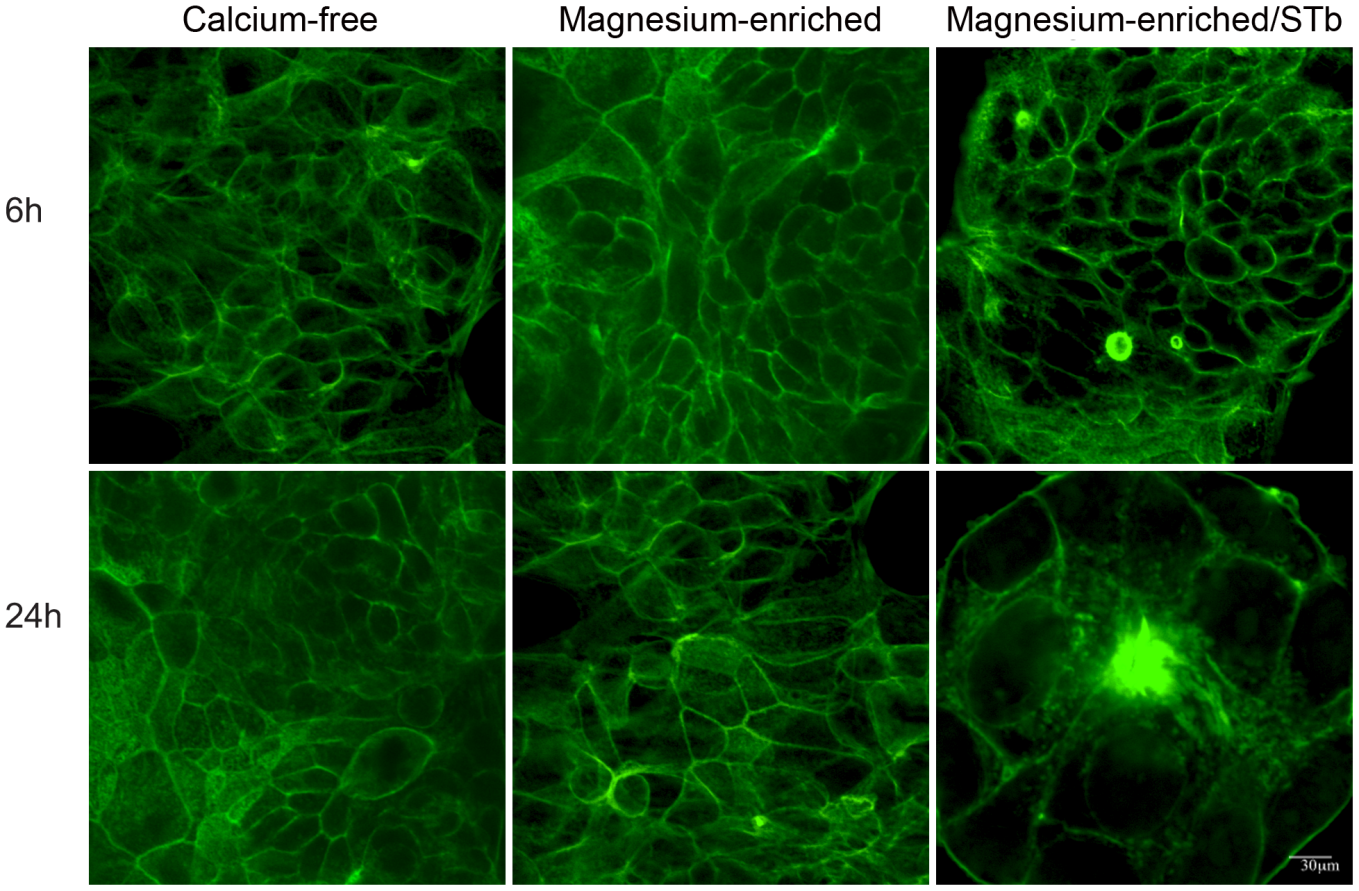
Effect of Mg^++^-enrichment on the rate of STb toxin activity. Cells grown in calcium-free and magnesium-enriched (1.8 mM) media were compared after 6 and 24 h. Confocal microscopy was used to analyze the distribution of actin filaments stained with FITC-phalloidin. Magnesium-enriched medium had no visible effect on the actin organization whereas in calcium-enriched medium STb provoked actin condensation after 24 h. In calcium-free medium, actin condensation was observed only after 24 h (Data not shown) Bar, 30 µm.

**Figure 8:**
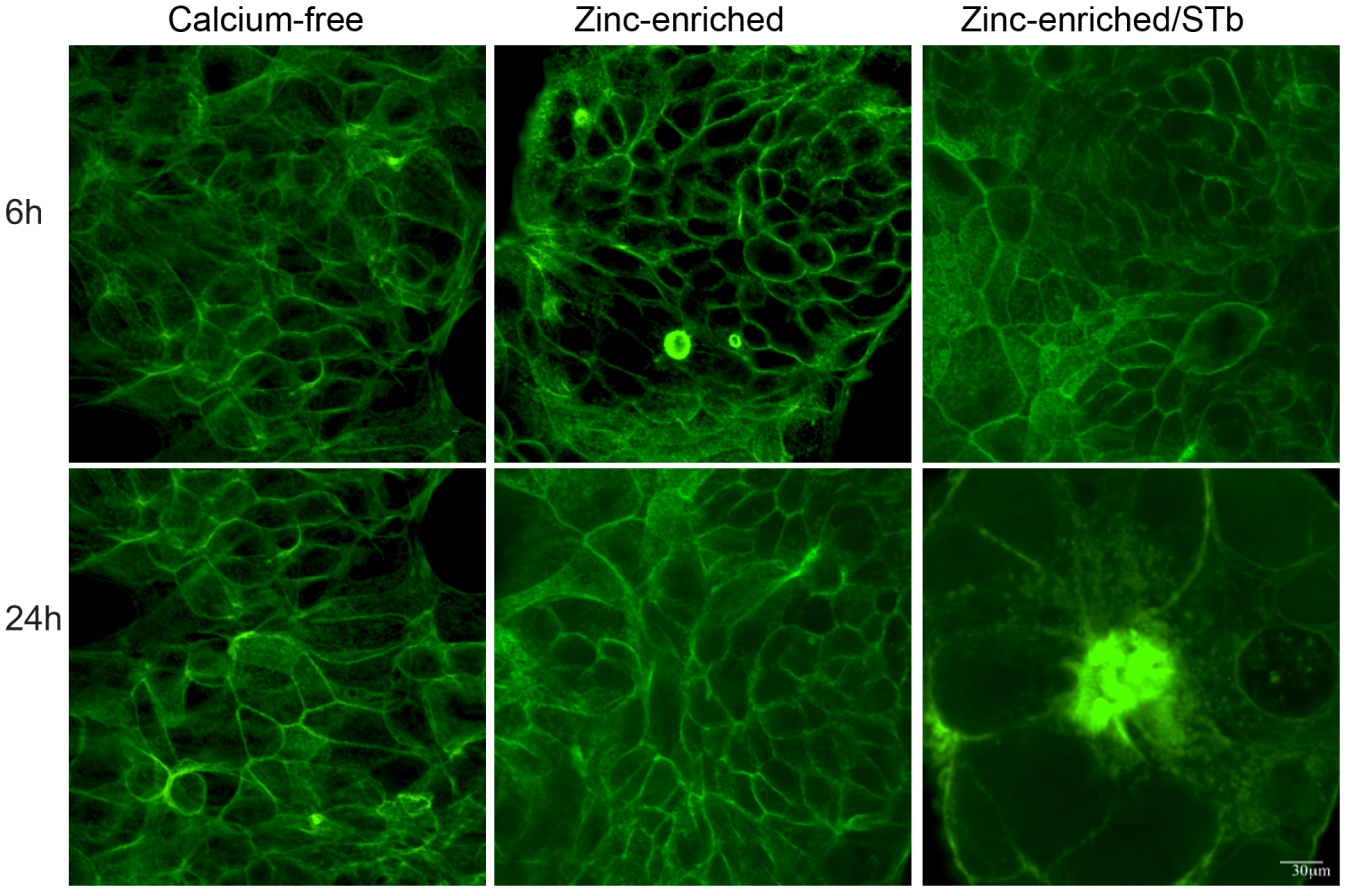
Effect of Zn^++^-enrichment on the rate of STb toxin activity. Cells grown in calcium-free and zinc-enriched (1.8 mM) media were compared after 6 and 24 h. Confocal microscopy was used to analyze the distribution of actin filaments stained with FITC-phalloidin. Zinc-enriched medium had no visible effect on the actin organization whereas in zinc-enriched medium STb provoked actin condensation after 24 h. In calcium-free medium, actin condensation was observed only after 24 h (Data not shown) Bar, 30 µm.

To assess the specificity of Ca^++^ on the rate of claudin-1 dislodgment and actin condensation, two divalent metal ions, Mg^++^ and Zn^++^ were compared to Ca^++^. As seen in Figure 5 and 6, the rate in redistribution of NP-40-soluble and -insoluble claudin-1 levels were shown to be Ca^++^ specific as neither Mg^++^ nor Zn^++^ could increase the rate of claudin-1 dislodgement. Nevertheless, changes in claudin-1 location and actin condensation in Mg^++^- and Zn^++^- enriched media were observed as described before in calcium-free medium after 24 h (Fig. 7 and 8).

### Phosphorylation state of claudin-1

**Figure 9:**
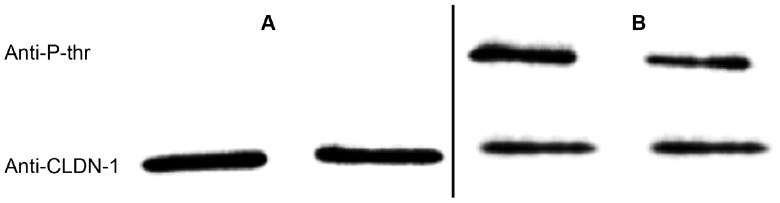
Phosphorylation state of claudin-1 as found in the membrane and cytoplasmic locations. (A) NP-40-soluble and (B) NP-40-insoluble fractions of STb-treated T84 cells. Left panel: untreated, right panel: STb-treated. Cell proteins extracted with NP-40 were migrated on a 12% acrylamide SDS-PAGE and immunoblotted with anti-phosphothreonine (Anti-P-thr). The amount of claudin-1 was adjusted to the same level in the various fractions using the anti-claudin-1 antibodies (anti-CLDN-1). The claudin-1 in NP-40-insoluble fraction is phosphorylated whereas in the NP-40-soluble fraction it is not phosphorylated. The amount of phosphorylated claudin-1 in the NP-40-insoluble was decreased in STb treated cells compared to control (p<0.001).

The phosphorylation state of claudin-1 in NP-40-insoluble (membrane location) and -soluble fraction (cytoplasmic location) was studied. For comparison purposes, claudin-1 levels were adjusted, in the various fractions, to the same level as can be observed with anti-claudin-1 (Fig. 9). In untreated T84 cells, threonine phosphorylation was observed in the NP-40-insoluble fraction using anti-phosphothreonine antibodies (Fig. 9B, left panel), while nonphosphorylated threonine was found in the NP-40-soluble fraction (Fig. 9A, left panel). Moreover, the level of phosphothreonine in claudin-1 was reduced in the NP-40-insoluble fraction after 24 h of exposure to STb toxin (Fig. 9B, right panel). Thus, STb toxin induces the dislodgment of claudin-1 from membrane to cytoplasmic location and this change correlated with the observation of non-phosphorylated claudin-1. The same results were observed using an anti-serine antibody (Data not shown).

## Discussion

A number of factors can be responsible for epithelial barrier dysfunction, including microbial infection. Enteric pathogens have developed strategies that induce the production of diarrhea in infected hosts, through disruption of intercellular TJs [45], [46]. Many studies have indicated that toxins can modulate the epithelial barrier by targeting junctional as well as cytoskeletal cell components and thus for some pathogens the changes facilitates invasion across the mucosal surface [47]. In this study, we investigated effects of STb toxin on claudin-1 location and phosphorylation levels in cultured T84 epithelial cells.

LT and STa toxin were recently shown to cause an increase in the epithelial cell permeability observed as a trans-epithelial resistance (TER) decrease and passage of dextran-FITC by disrupting TJs of T84 cells [30]. We thus compared the cellular NP-40 detergent partitioning and the state of phosphorylation of claudin-1 in epithelial T84 cell line following STb treatment. This cell line display distinct barrier characteristics (i.e., T84 cells form a very tight monolayer with high TER and low permeability to uncharged molecules) [31], [33].

We first assessed claudin-1 location in T84 cells by immunostaining and confocal imaging. Claudin-1 and occludin has been demonstrated before to be apically located sealing the TJs [31], [33]. It has also been suggested that claudin-1 contribute to cell adhesion [48]. In our study, claudin-1 was recovered in both the NP-40-soluble and-insoluble fractions of the cell extracts (Fig. 2 a, b). Since the TJ strands are assumed to be resistant to detergent extraction [49], these findings indicates the existence of claudin-1 both as a junctional complex and as a soluble pool of proteins. The distribution of claudin-1 in two distinct subcellular pools had been reported earlier [50].

Mechanisms underlying the perturbation of the epithelial barrier are numerous. Some compounds directly interact with single TJ proteins [51], whereas others disturb barrier function by a general modification of TJ strands [52]. Reorganization of the actin ring also results in loss of tight junction integrity [53]. STb was previously found to affect the actin ring of T84 cells [31]. The authors also reported shortening of the actin filaments and appearance of actin condensation after treatment with STb toxin. This is consistent with our results (Fig. 2) where shortening of the filaments was observed. The lack of reorganization of the actin ring suggests that STb does affect TJs via a change in actin architecture. The relevance of the changes in actin filaments with regard to TJs integrity remains to be established. Claudin-1 interacts with actin through proteins such as ZO-1 and ZO-3 and we have shown in a previous study that the increase in paracellular permeability resulting from STb activity is associated with fragmentation of ZO-1 [31].

The effect of STb on TJ proteins was examined in an attempt to clarify the mechanisms that underlies the observed changes. The loss of claudin-1 from membranes in STb-treated T84 cell monolayers indicates a loss of TJ integrity and therefore changes in barrier properties. Western blotting of T84 cell fractions provided further evidence for a change in subcellular location of the TJ protein claudin-1. The claudin-1 content of the membrane fraction decreased with STb treatment in a time-dependent manner (Fig. 1) while the claudin-1 content of the NP-40-soluble fraction increased with time, indicating that claudin-1 translocate from the membrane to the cytosol in response to treatment with STb. This shift of claudin-1 into the soluble fraction had been observed in Caco-2 cells following ATP depletion (15, 21).

The observed effect of STb on claudin-1 delocalization is likely contributing to the pathogenesis of ETEC by allowing the passage of electrolytes and water through the paracellular space. As shown in our study, in absence of Ca^++^ this delocalization happened after 24 h but in presence of this metal ion we could observe a similar effect after only 6 h (Figure 3 and 4). Opening of TJs as an early event, we believe, is responsible at least in part for the fluid secretion resulting from STb intoxication. Dreyfus et al. (1993) examined the effect of STb on the internal Ca^++^ concentration of transformed and primary cells of different tissues and animal species. They suggested that STb opens a GTP-binding regulatory protein-linked receptor-operated Ca^++^ channel in the plasma membrane. Beyond these studies, nothing is known of the relation between STb, Ca^++^ and TJ proteins. Our results suggest that STb induces a rapid time-dependent toxicity by translocation of claudin-1 from membrane to a more soluble form.

The increased calcium levels are also thought to regulate phospholipases (A2 and C) that release arachidonic acid from membrane phospholipids, leading to the formation of intestinal secretagogues PGE_2_ and 5-HT, which mediate water and electrolyte transport out of intestinal cells [54]. In our study, the important role of calcium was confirmed where by adding the calcium to the culture medium STb increased the rate of disruption of the barrier integrity. Others divalent cations had no significant effect on STb toxicity.

It has been proposed that claudins can be regulated by PKC-mediated phosphorylation, modulating barrier function in the cells [33]. Thus, in Caco-2 cells, inactivation of PKC-Φ reduced phosphorylation of claudin-1 and decreased the membrane to cytosolic distribution of the claudin-1 [55]. In ovarian cancer cells, phorbol ester-mediated PKC activation induced phosphorylation of claudins and decreased barrier function [56], and in human epidermal keratinocytes, formation of TJs was suggested to be regulated by a PKC-induced phosphorylation of claudin-4 [57]. A previous study had shown binding of STb to its receptor is associated with the uptake of Ca^++^ into the cell, activating PKC, which through phosphorylation activates CFTR [58]. Because STb activates PKC and that STb opens a GTP-binding regulatory protein-linked receptor-operated Ca^++^ channel in the plasma membrane [10], we can hypothesize that PKC signalling pathway may be involved in TJs dysfunction as well as cytoskeletal changes. In T84 untreated cells, phosphorylated claudins-1 was found only in NP-40 insoluble fraction, whereas in NP-40 soluble fraction, claudin-1 was not phosphorylated. Since STb induces a major decrease in the apical distribution of claudin-1 and a reduction in the barrier function [31], a change in the phosphorylation state might be the mechanism by which STb acts indirectly.
