# Correction: Escherichia coli STb Enterotoxin Dislodges Claudin-1 from Epithelial Tight Junctions

**DOI:** 10.1371/journal.pone.0118983

**Published:** 2015-03-03

**Authors:** The PLOS ONE Staff

Fig. 6 and Fig. 8 are incorrect. The authors have provided corrected versions here.

**Fig 6:**
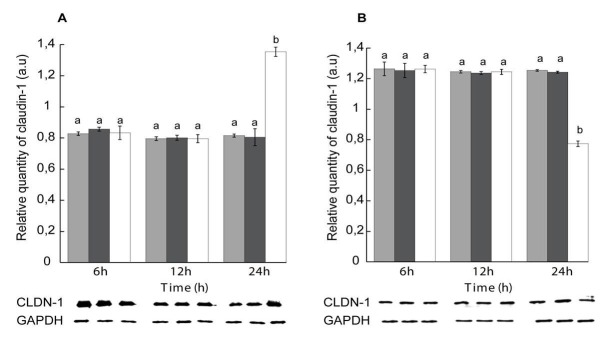
Effect of Zn^++^ -enrichment on claudin-1 displacement rate. (A) NP-40-soluble and (B) NP-40-insoluble fractions. Gray: calcium-free medium, black: Zn^++^-enriched medium, white: Zn^++^-enriched medium treated with STb for 6, 12 and 24 h. Lower panel: Immunoblot showing claudin-1 and GAPDH used to evaluate their relative amounts. NP-40 cell extracted proteins were separated on a 12% acrylamide SDS-PAGE and immunoblotted with anti-claudin-1 and anti-GAPDH antibodies. The calcium-free medium was Zn^++^ -enriched (1.8 mM). There was no significant difference in claudin-1 dislogment rate under Zn^++^-enriched condition compared to calcium-free medium. After 24 h, claudin-1 dislogment was observed as seen before in calcium-free medium (n = 3) (p<0.001). CLDN-1: claudin-1, GAPDH: Glyceraldehyde 3-phosphate dehydrogenase. Letters on top of the bars when different indicates a statistical difference between the treatments.

**Fig 8:**
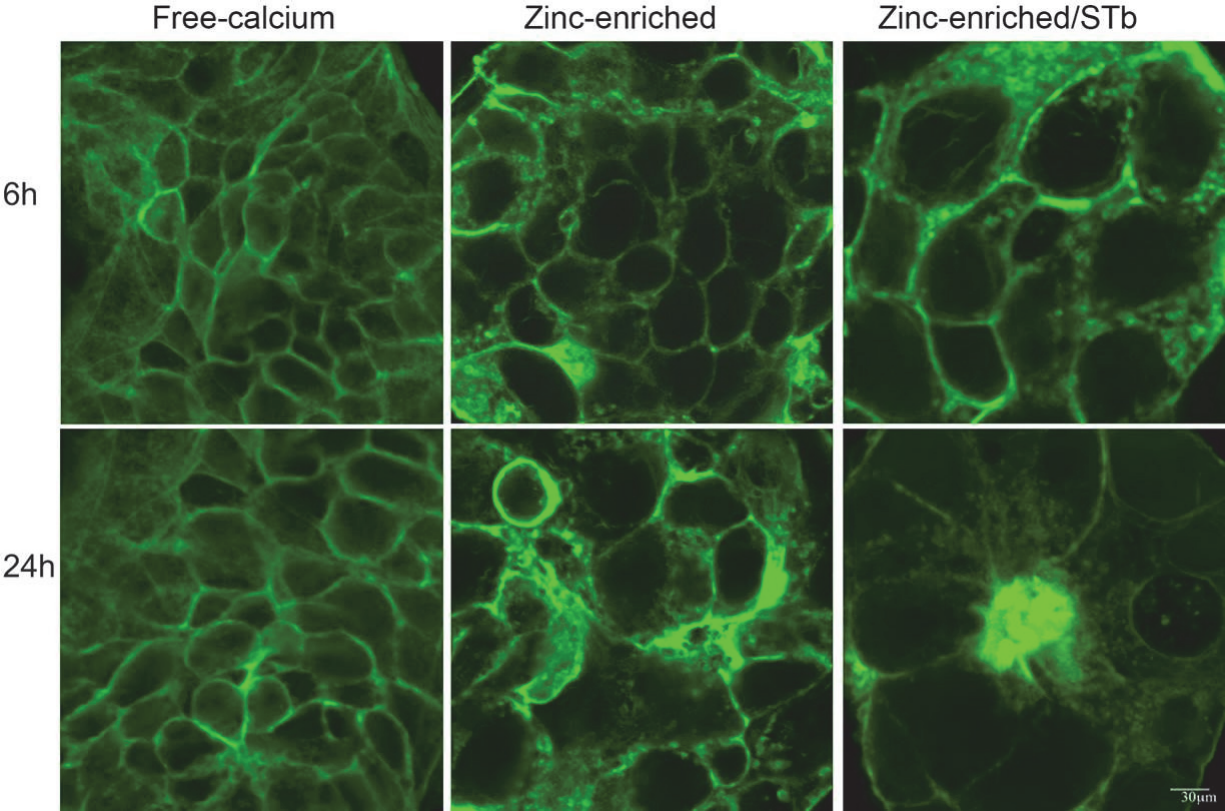
Effect of Zn^++^-enrichment on the rate of STb toxin activity. Cells grown in calcium-free and zinc-enriched (1.8 mM) media were compared after 6 and 24 h. Confocal microscopy was used to analyze the distribution of actin filaments stained with FITC-phalloidin. Zinc-enriched medium had no visible effect on the actin organization whereas in zinc-enriched medium STb provoked actin condensation after 24 h. In calcium-free medium, actin condensation was observed only after 24 h (Data not shown) Bar, 30 μm.
